# Mental health training programs for non-health professionals and volunteers working with asylum-seekers and refugees: scoping review

**DOI:** 10.1590/1980-220X-REEUSP-2022-0447en

**Published:** 2023-08-25

**Authors:** Luísa Micaela Teixeira-Santos, Filipa Isabel Quaresma Santos Ventura, João Artur Oliveira Santos, Inês Franco de Almeida, Wilson Correia Pinto de Abreu

**Affiliations:** 1 Universidade do Porto, Instituto de Ciências Biomédicas Abel Salazar, Porto, Portugal.; 2 Centro de Investigação em Tecnologias e Serviços de Saúde, Porto, Portugal.; 3 Escola Superior de Enfermagem de Coimbra, Unidade de Investigação em Ciências da Saúde: Enfermagem, Coimbra, Portugal.; 4 Unidade de Saúde Local de Matosinhos, Porto, Portugal.; 5 Escola Superior de Enfermagem do Porto, Porto, Portugal.

**Keywords:** Refugees, Mental Health, Education; Nursing, Refugiados, Salud mental, Educación, Enfermería

## Abstract

**Objective:**

To identify and describe the mental health training programs for non-health professionals and volunteers who work, have worked, or would work with asylum seekers and/or refugees.

**Method:**

Scoping review following JBI methodology. Search carried out in MEDLINE, CINAHL, ERIC, SCOPUS, PsycINFO, Psychology & Behavioral Sciences Collection, RCAAP, ProQuest, and websites of Clinical Trials, UNHCR, International Organization for Migration, WHO, Save the Children, International Migration, Integration and Social Cohesion in Europe, and International Federation of Red Cross and Red Crescent Societies. Studies written in English, Portuguese, French, Spanish and Swedish.

**Results:**

Of the 8954 articles identified, 16 were included reporting on 11 training programs: Mind-Spring, PM+, MHFA, Cognitive-Behavioral Training for Community and Religious Leaders, EmpaTeach, Suicide Prevention Education Program, Teaching Recovery Techniques, Handbook for Teachers of Vietnamese Refugee Students, PFA, Psychosocial support of volunteers and CBP&MHPSS.

**Conclusion:**

Training programs from scientific literature focus on mental health disorders, while non-governmental organizations’ documents focus on resilience and self-care. The current mental health training programs might be insufficient.

## RESUMEN

**Objetivo:**

Identificar y describir los programas de formación en salud mental para profesionales no sanitarios y voluntarios que trabajan, han trabajado o gustarían de trabajar con solicitantes de asilo y/o refugiados.

**Método:**

Revisión de alcance según la metodología JBI. Búsqueda realizada en MEDLINE, CINAHL, ERIC, SCOPUS, PsycINFO, Psychology & Behavioral Sciences Collection, RCAAP, ProQuest, y sitios web de ClinicalTrials, ACNUR, Organización Internacional para las Migraciones, OMS, Save the Children, Migración Internacional, Integración y Cohesión Social en Europa, y Federación Internacional de Sociedades de la Cruz Roja y de la Media Luna Roja. Estudios escritos en inglés, portugués, francés, español y sueco.

**Resultados:**

De los 8954 artículos identificados, se incluyeron 16 que informan sobre 11 programas de capacitación: Mind-Spring, PM+, MHFA, Capacitación cognitiva conductual para líderes comunitarios y religiosos, EmpaTeach, Programa de educación para la prevención del suicidio, Enseñanza de técnicas de recuperación, Manual para maestros de Estudiantes refugiados vietnamitas, PFA, apoyo psicosocial de voluntarios y CBP&MHPSS.

**Conclusión:**

Los programas de formación en la literatura científica se centran en los trastornos de salud mental, mientras que los documentos de las organizaciones no gubernamentales se centran en la resiliencia y el autocuidado. Los actuales programas de formación en salud mental pueden ser insuficientes.

## INTRODUCTION

The world as we know is changing, especially due to worldwide migrations. As a result of wars, violence, persecution, human rights violations, and events that seriously disturb public order, 89,3 million individuals worldwide were forcibly displaced by the end of 2021^(1)^. Of the 89,3 million people, 27,1 million are refugees, 53,2 million are internally displaced people, 4,6 million are asylum seekers^(2)^. Almost 70% of refugees came from the Syrian Arab Republic (6,8 million); Venezuela (4,6 million); Afghanistan (2,7 million); South Sudan (2,8 million); and Myanmar (1,1 million). The low- and middle-income countries hosted 83% of refugees, being Turkey the host country that receives the largest number of refugees worldwide (3,8 million), followed by Colombia (1,8 million), Uganda (1,5 million), Pakistan (1,5 million) and Germany (1,3 million)^(1,2)^.

In the last years, the forcibly migratory fluxes have been a concern to the European Union as the number of people seeking protection in Europe has grown considerably. From 2014 until the end of 2021, Italy, Cyprus, Malta, Greece, and Spain received 2,300,881 million sea and land arrivals^(3)^. These data focus on forced displaced people by the end of 2021. Along with the war in Ukraine in early 2022, which caused until now 7 million people to be internally displaced and 6 million people refugees, the statistics on influxes of European migration are dramatically changing^(1)^.

Forcibly displaced people are obligated to abruptly leave all belongings and often their family members to seek international safety and protection. The loss of material resources (e.g., house, clothing, belongings), identity references (e.g., social and cultural relations), as well as adequate access to essential care and resources such as health and education, are factors of vulnerability in mental health^(4,5)^. Forced displacement is increasing and several mental health studies are being conducted to understand the impact of this situation on people’s mental health. A recent systematic review of psychiatric disorders in refugee and internally displaced persons after forced displacement, including 38 studies that provided data from 39,518 participants from 21 countries, show that participants suffer from post-traumatic stress, depression, and anxiety disorders^(6)^. Mental health disorders are linked with pre-, ongoing, and post-migration situations and involve traumatic events, such as violence, separation, sexual abuse, trafficking, harassment, and lack of basic needs^(7,8,9)^. The lack of basic needs is not only present in the country of origin. As a result of some countries’ political arrangements, asylum seekers are getting stopped at the borders. For example, if asylum seekers arrive in Europe, they must wait years for the refugee legal status to have the right to get out of a refugee camp/shelter/reception centre, most of them with inhumane conditions^(10)^.

To maintain safety, well-being and safeguard the asylum seekers’ rights, the United Nations High Commissioner for Refugees (UNHCR) works in partnership with 900 entities, most of them Non-Governmental Organizations (NGOs)^(11)^, which are mainly composed of civil society volunteers^(12)^. Motivational factors drive volunteers to spend their time working on helping others. Yet they have the major challenge of dealing with the suffering of asylum seekers and refugees (AS&R) as they listen about the serious traumatic trajectories in their pre, during, and post-migration period on a daily basis^(13)^. Volunteers working with AS&R in a chronic stress environment may increase their vulnerability to adverse consequences, such as anxiety, burnout, and depressive feelings, over-involvement with AS&R, callousness, apathy, self-destructive behaviour, interpersonal conflict, and secondary traumatic stress^(14,15,16)^. Several studies show that volunteers’ psychological distress can vary depending on previous training^(17,18,19)^. Psychologically trained refugee-helpers had lower burnout values and somatic symptoms when compared with untrained aid workers^(20)^.

Psychological training is relevant not only for volunteers’ mental health preparation and safety but also to provide better and adequate care for AS&R. Improving mental health competencies, skills, and knowledge of volunteers, non-health professionals, i.e., who do not have a mental health background education, is likely to have a positive impact in the AS&R’ health. Mental health training for volunteers working with AS&R has shown evidence of empowering them to make earlier and correct decisions about prevention, early detection, and appropriate referral for specialized mental care, reducing stigma and discrimination, and improving AS&R rights^(21,22)^. The UNHCR defends that interventions towards the promotion of psychosocial support with AS&R can be provided by non-specialized mental health volunteers, yet they must be trained and supervised^(22)^. This evidence reinforces the importance of mental health training programs for volunteers in this fieldwork.

The scientific research with volunteers and their work with AS&R is slowly increasing^(21)^. A systematic overview to understand the kind of mental health competencies training available to prepare volunteers for their work with AS&R is demanded. Mental health competence is understood as the ability (attitudes, knowledge, skills, and behaviours)^(23)^ to effectively promote prevention, care, treatment, and advocacy for mental health. This competence requires knowledge to protect their self-mental health; to recognize people’s suffering, based on their cultural competencies; to provide basic psychosocial support to vulnerable populations during their overwhelming life transitions; and an empowerment skill to refer people for specialized mental care or to mental health professionals^(24)^.

Nurses are vital in the promotion of health and health literacy^(25,26)^, and they are the key factor in the health responses of these populations^(27,28)^. Therefore, this scoping literature review aims to identify and describe the mental health training programs for non-health professionals and volunteers who work, have worked, or will work with AS&R regardless of the context, i.e., mental health training programs and courses available for the civil society members, who do not have a health or mental health background but need to develop those skills to deal with AS&R. This review is fundamental to understand the people conducting these training programs, the places where the training takes place, and the educational domains and strategies that are used.

## METHOD

### Design of Study

This scoping review was conducted according to the JBI methodology for scoping reviews^(29,30)^, following an *a priori* published protocol which describes the methodological procedures used^(24)^.

The PCC (Population, Concept, and Context) mnemonic^(29,30)^ was used. P representing studies with or addressed or designed for participants aged ≥18 years, volunteers, and non- health professionals, without mental health training, independent of their educational level, who had work, were working, or would work with asylum seekers or/and refugees. C represented studies reporting on the training of adults in mental health competencies. C was considered to be any environment where the study was developed. The review aimed to respond to the main research question: What are the mental health training programs that have been used in the preparation of non-health professionals and volunteers who do not have mental health training to work with AS&R?

**Chart 1:**
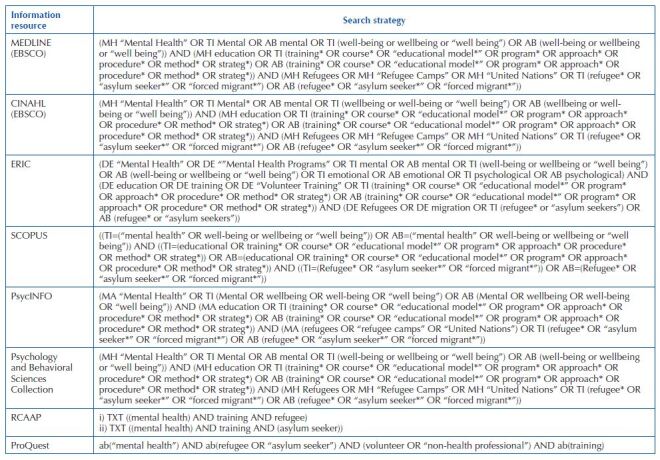
Scoping review databases’ search strategy – Porto, Portugal, 2021.

An initial search was carried out in MEDLINE (PubMed) and CINAHL (EBSCO) databases to analyse the terms used to describe the articles relevant to the study. Then, a full search strategy described in Chart 1 with terms adapted for each source, was carried out in the MEDLINE (EBSCO), CINAHL (EBSCO), ERIC, SCOPUS, PsycINFO, Psychology & Behavioral Sciences Collection, and in the grey literature databases The Scientific Open Access Repository of Portugal (RCAAP), and ProQuest. In the scoping review protocol^(24)^, the authors identified as a source of grey literature the OpenGrey database. However, OpenGrey was discontinued in 2021. To be able to integrate international grey literature in this review, the authors came to the consensus of including the ProQuest database. The scoping review search also included the websites of ClinicalTrials, UNHCR, International Organization for Migration (IOM), WHO, Save the Children, International Migration, Integration and Social Cohesion in Europe (IMISCOE), and International Federation of Red Cross and Red Crescent Societies (IFRC). The NGO websites listed as sources of information were provided by a WHO Guidance Note that identifies them as NGOs with developed work on protecting and supporting the mental health and psychosocial well-being of refugees, asylum seekers, and migrants^(31)^. In the NGO websites, the search was made through the search bottom, in the documentation section, with the common words “mental health training” or “training”. On the ClinicalTrials website, the search included six combinations of common words: i) mental health training AND refugees; ii) mental health training AND asylum seekers; iii) mental health training AND refugee volunteers; iv) mental health AND asylum seekers volunteers v) mental health AND refugee workers; vi) mental health AND asylum seekers workers.

### Selection Criteria

The review considered studies reporting training or training’ protocols of mental health competencies for adults, non-health professionals and volunteers without mental health training, who had work, are working, or will work with asylum seekers or/and refugees without the context restrictions, available in English, Swedish, Portuguese, Spanish, and French. For this review it was considered for inclusion primary studies, quantitative, qualitative, mixed- and multi-method study designs, reviews, protocols, conference abstracts and text opinion papers with relevant information until December of 2021. In addition, several relevant websites were searched to identify information that might not be available in scientific databases, such as the websites of reputable NGOs, as described in the previous section. Specially from these sources the participants could be named as field workers, which is a common designation for someone who works in the humanitarian field.

### Data Collection

Data collection took place from December 15^th^ to December 31^st^ of 2021. Initially, all the articles found were uploaded into EndNote^TM^ X8 software, and the duplicates were removed. After this process, the studies were uploaded into the Rayyan software to proceed with the initial screening of titles and abstracts by two independent reviewers. The full text of selected citations was assessed in detail against the inclusion criteria by two independent authors. At each stage, the doubts about the article’s selection were discussed by reviewers and ultimately decided by the principal investigator. The selection process was guided by the Preferred Reporting Items for Systematic Reviews and Meta-Analyses extension for Scoping Reviews (PRISMA-ScR) checklist^(32)^.

### Data Analysis and Treatment

Two independent authors extracted data from the sources included in the scoping review using a data extraction tool developed by the reviewers presented in the published scoping review protocol^(24)^. The principal investigator gathered and compiled in tables all the extracted information according to the developed tool. All members of the team were involved in the development of the data extraction form. The form was piloted test independently by two researchers (LTS, FV). The form was then revised in consultation with an experienced reviewer (WA) to promote consistent and reliable extraction. The extracted information was analysed according to the following categories: study identification (ID); reasons for inclusion or exclusion; characteristics of study population/paper, participants, settings, educational mental health domains, and the strategies that were used for the training; and a category about research methods used in the study/paper.

Data extraction was performed independently by five researchers (LTS, FV, JS, LT, IA) and reviewed by a senior researcher with extensive review experience (WA). Conflicts were discussed, and where necessary, a third author was consulted (LTS) in consultation with an experienced reviewer (WA).

### Ethical Aspects

The reliability and fidelity of the information extracted from the selected publications were ensured through proper referencing and rigour in data treatment and presentation. This review was conducted under a PhD project named APT4U2, which was approved by the Ethics Committee of the Health Sciences Research Unit: Nursing (no. 0 P742 12/2020).

## RESULTS

**Figure 1:**
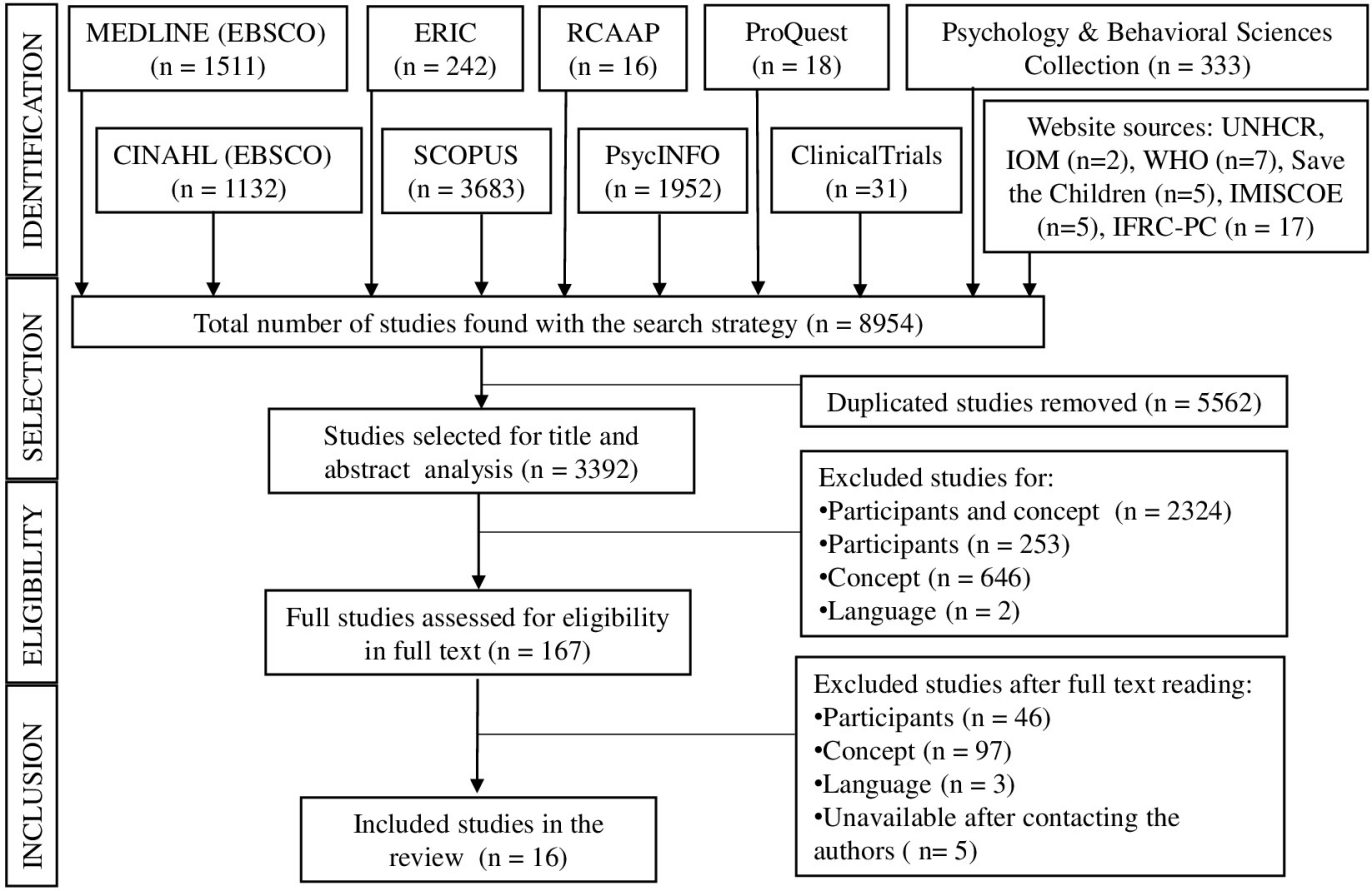
Scoping review structure flowchart. Porto, Portugal, 2022.

The search strategy identified a total of 8,954 publications. After excluding the 5,562 duplicates, 3,392 studies were selected for title and abstract analysis. A total of 167 were selected for full reading analysis, with the remaining 16 articles included in the review, as shown in Figure 1.

**Chart 2:**
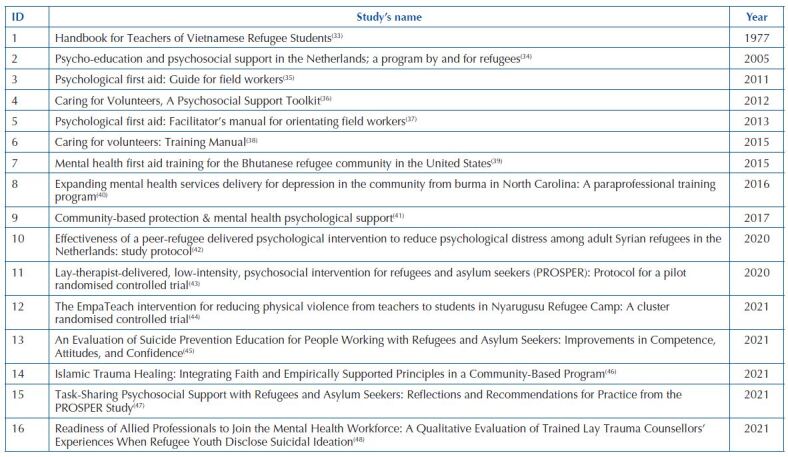
Studies included in the scoping review – Porto, Portugal, 2022.

The 16 studies included in the scoping review are presented in Chart 2.

All the articles included were published in English, and those from the website sources^(35–38,41)^ were translated into at least three more languages. Eleven articles were taken from scientific databases^(33,34,39,40,42–48)^, six of which were experimental studies carried out in the Netherlands^(34)^, United States of America^(39,40)^, Tanzania^(44)^, Australia^(45)^, and Sweden^(48)^. Of the other five studies, two were randomized controlled trial protocols to be carried out in the Netherlands^(42)^, and the United Kingdom^(43)^, and three studies described an intervention without results from implementation^(33,46,47)^. Of the five included from website sources, two are from OMS^(35,37)^, two from the Psychosocial Centre of the IFRC^(36,38)^, and one from UNHCR^(41)^. All of these reported interventions for training in mental health competencies to work with AS&R but did not describe examples of implementation.

As for the year of publication, the first study that was made available on the databases concerning this subject dates back to 1977^(33)^. The number of scientific publications on the topic has increased since 2015, and 2021 was the year with the highest number of records (n = 5)^(44–48)^.

Answering the main question of this study, different programs were identified as training programs in mental health competencies for lay people working with AS&R, namely Mind-Spring^(34)^, Problem Management Plus (PM+)^(42,43,47)^, Mental health first aid (MHFA)^(39)^, Cognitive-Behavioral Training (CBT) for Community and Religious Leaders^(40)^, EmpaTeach^(44)^, Suicide Prevention Education Program^(45)^, Teaching Recovery Techniques (TRT)^(47)^, and a Handbook for Teachers of Vietnamese Refugee Students^(33)^. From the organization’s website sources, five documents reported on interventions on training in mental health competencies for field workers to work in crises situations. Although not specific to work with AS&R, the included documents present case scenarios, activities or the description of crises situations involving situations with AS&R, namely: Psychological First Aid (PFA)^(35,37),^ Psychosocial support of volunteers^(36,38)^, Community-based protection and Mental health psychological support (CBP & MHPSS)^(41)^.

**Chart 3:**
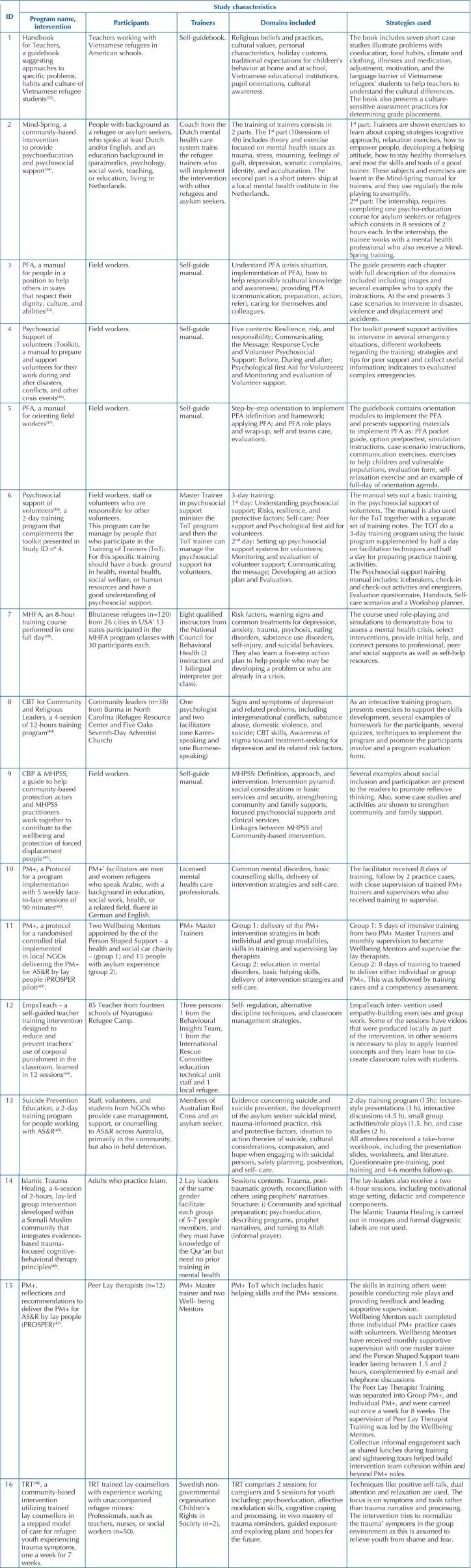
Studies included in the scoping review – Porto, Portugal, 2022.

The studies’ characteristics regarding the trainers, educational domains and strategies used are presented in Chart 3.

## DISCUSSION

AS&R experience mental health challenges that reduce their well-being not only related to the experiences in their country of origin but also when in transit and with the reception on arrival, including accesses to housing or healthcare. The contact made between AS&R and the healthcare system is often crisis-driven and mediated through NGOs, whose staff lack knowledge and skills in the management of distress^(4,43)^.

To respond to the shortage of health professionals in the world^(49)^, especially in low- and middle-income countries where conflicts, disasters, and poverty is more common, thereby leading to an increase in AS&R, the WHO proposes professional training of lay counsellors to provide mental health interventions^(50)^. Additionally, the Inter-Agency Standing Committee recommended guidelines for emergency relief efforts and proposed that mental health interventions could be delivered by trained, nonprofessional community members^(51)^. In light of the results of this review, the number of scientific studies researching on programs and/or interventions to train mental health competencies of the volunteers, which includes community members, and NGO staff, with no health or mental health background to work with AS&R is insufficient. Most are protocols focused on specific mental health problems or cultural and religious backgrounds, incapable of being used in several contexts or self-administrated without control of the acquired knowledge. However, the number of articles about the topic has been increasing, especially since 2015. This increment might be related to the exponential increase of forcibly displaced people since 2015, the highest since World War II^(52)^. Altogether these situations led to a forced exodus of populations to neighbouring countries.

The investment in mental health training of community workers or volunteers and staff with no background in the health domain is aligned with the principle that non-specialists can help to increase access and effectively provide mental health interventions in low-resource communities^(53)^. As shown, all the included studies in this review aimed to guide the volunteers on how to provide humane, supportive, and practical help to adults and children AS&R experiencing crisis events. Most of the articles are designed for lay providers regarding their educational backgrounds. Two articles are specific for teachers, one is a handbook to guide American teachers to deal with Vietnamese refugee children^(33)^, and another one is a self- guided teacher training intervention designed to reduce and prevent teachers’ use of corporal punishment in the refugee camps’ classrooms in Tanzania^(44)^. The manuals of WHO^(35,37)^, IFRC^(36,38)^, and UNHCR^(41)^ are designed for field workers, i.e., people who support others during or immediately after extremely stressful events. The remaining studies published in the scientific databases focused mainly on training people with refugee^(34,39,40,42,43,47)^ or asylum seekers’ backgrounds^(34)^, NGO volunteers, and professionals working in the incoming countries^(43,44,46)^. Although lacking specifications regarding the educational background, some of the studies indicated that participants should preferably be psychologists, social workers, teachers, nurses, leaders, volunteers’ managers, or workers in related fields.

Regarding the domains included, the WHO^(35,37)^, IFRC^(36,38)^, and UNHCR^(41)^ manuals focused essentially on PFA^(35,37)^ and MHPSS^(41)^ to help people in crises and psychosocial support for volunteers approaching resilience, risks, and self-care^(36,38)^. The articles from the databases focused mainly on common mental health disorders^(42,43)^ such as trauma^(34,40,45,46,48)^, stress, mourning, feelings of guilt, somatic complaints, identity and acculturation^(34)^, depression^(34,39,40)^, psychosis, eating disorders, substance use disorders^(38,40)^, self-injury, and suicidal behaviours^(39,40,45)^. The PM+, which is an individual psychological help for adults developed by the WHO, was modified to be an evidence-based psychosocial intervention delivered by lay staff to help AS&R with basic counselling skills, delivery of intervention strategies and self-care^(43,47)^. All articles and documents included in this review broadly agree that psychological support can be facilitated by lay people for vulnerable populations, such as AR&R, which in most cases can be people with previous asylum experience or people with high interest and willingness to help these populations. However, due to the cultural background of each population, they all agree that any programs that are considered a model need to be adapted appropriately to the local context and the culture of the people who will be assisted by volunteers. Although most lay facilitation programs address similar domains, the time devoted to training is quite diverse. Lay-facilitators’ programs duration was found to last eight hours in a single day^(39)^, to a two-day training of 15h^(45)^, six-sessions of two hours^(46)^, four-session of 12-hours^(40)^, five weekly face-to- face sessions of 90 minutes^(42)^, 10 sessions of four hours each, including internship^(34)^, three-day training^(38)^, and seven weeks without specification of hours^(48)^.

The current findings may be important for health authorities, policymakers, and other stakeholders planning to provide mental health training to NGO volunteers and staff in the incoming countries or even in humanitarian settings. In particular, for people preparing others to work in humanitarian settings, which is increasing worldwide due to forced migrations, the use of lay mental health providers could be a valuable, first-tier psychological support service for people in underserved communities such as AS&R.

The heterogeneity of the interventions used in the included studies is both a strength and a weakness. On the one hand, the diversity in the type and length of training shows both the investment that is being made to respond to emerging local needs and a concern to train the volunteers and the staff of community helpers under supervision to make the provision of support to a greater number of people in need possible. On the other hand, this diversity challenges the results’ interpretation regarding the training programs and their comparison. In some of the studies, the training is self-guided by manuals or handbooks with no facilitator, which could lead to a misunderstanding of contents. Other studies did not describe the supervision conditions as part of or after the training.

Further research is needed on mental health training of non- health professionals and volunteers that are in places where mental health needs outrun professional resources s (e.g., refugee camps, shelters, and reception centers), whether in humanitarian crisis contexts or the reception of refugees in host countries. Furthermore, research should explore the psychological impact of becoming a lay facilitator and the influence of the mental health training programs on their well-being and the well-being of the AS&R they assist. This is especially important in the case of interventions recruiting members from the same community that has been exposed to similar experiences. In addition, it would be interesting to evaluate the impact of mental health training programs on the relationships between staff or volunteers and AS&R and the acculturation process.

Regarding the limitations of the study, some articles or documents did not provide a satisfactory description of the intervention or the participants’ characteristics. Furthermore, data about settings, hours, strategies of training sessions, and the kind of participants’ supervision during or after the training was also absent. Additionally, the studies included did not specifically address work with AS&R; they were rather included as they presented training to work in the humanitarian field or crisis situations by using examples of scenarios with displaced people.

Although the programs in mental health competencies for non-health professionals or volunteers to work with AS&R are scarce, we identified eleven training programs or interventions with this purpose, some of them being self-guided sources of training. Even though lay people have a promising role to play in assisting the AS&R and referring them to specialized care, the number of mental health training programs to train the volunteers is insufficient. In addition, the fact that working with AS&R is often voluntary work means that prior training is not required, and this could be the reason why greater and better investment is not made in training those who take care of the AS&R. This review demonstrates the need to invest in the development and implementation of mental health training programs in which nurses can play a vital role. As the ICN highlights, nurses are the key to caring for migrant populations^27^. Nursing care is indispensable for the easement of human distress and for the promotion of comfort and coping. Nurses also have an essential role in advocating for policies that will enhance AS&R’s access to health and mental health care and address barriers irrespective of AS&R status. By becoming aware of the existing challenges regarding access to mental health professionals and care, nurses’ intervention, support, and training of those caring for AS&R can help in different ways. In mental health training of non-health professionals and volunteers working with AS&R, mental health nurses, as qualified mental health educators, stand out for their ability to develop, coordinate, and implement mental health training programs which are activities aligned with the mental health and psychiatric field of nursing^(25)^. Mental health training provided by mental health and psychiatric nurses encourages the empowerment of the non-health professionals and volunteers to deal with the AS&R’s overwhelming life transitions; enhances the ability to refer people who need specialized mental health care; makes possible earlier care for those who have the luck of having someone trained who can help and recognized their needs; and promotes the non-health professionals and volunteers’ self-mental health care.

## CONCLUSION

Sixteen articles aboard eleven training programs to promote mental health competencies training: Mind-Spring, PM+, MHFA, Cognitive-Behavioral Training for Community and Religious Leaders, EmpaTeach, Suicide Prevention Education Program, Teaching Recovery Techniques, Handbook for Teachers of Vietnamese Refugee Students, PFA, Psychosocial support of volunteers and CBP&MHPSS.

Training programs from scientific literature focus mainly on mental health disorders, while non-governmental organizations’ documents focus on resilience and self-care. The eleven training programs included in this review might not be sufficient to meet the training needs of non-health professionals and volunteers working with AS&R. It is important to highlight that some studies are focused on specific cultural backgrounds or religious beliefs or based on the capacity of self-education of each person, which can contribute to lack of training to work in several countries, and to misinterpretation of the training concept as information available in the self-guided manuals. NGO volunteers and professionals with no educational background in the health domain need comprehensive training to deal with others’ mental health suffering without jeopardizing their mental health. This implies recognizing signs and symptoms of mental health problems that allow them to refer people to specialized care, understand how cultural background influences the suffering manifestations, and acquire strategies to take better care of themselves.

To fill this gap, mental health nurses should be on the front line to help people improve their mental health competencies to work with AS&R attending their cultural backgrounds.
